# A Volumetric Analysis of the ^1^H NMR Chemical Shielding in Supramolecular Systems

**DOI:** 10.3390/ijms22073333

**Published:** 2021-03-24

**Authors:** Jiří Czernek, Jiří Brus

**Affiliations:** Institute of Macromolecular Chemistry, Czech Academy of Sciences, Heyrovsky Square #2, 16206 Prague, Czech Republic; brus@imc.cas.cz

**Keywords:** proton NMR, chemical shielding, antiaromaticity, GIAO, B3LYP

## Abstract

The liquid state NMR chemical shift of protons is a parameter frequently used to characterize host–guest complexes. Its theoretical counterpart, that is, the ^1^H NMR chemical shielding affected by the solvent (^1^H CS), may provide important insights into spatial arrangements of supramolecular systems, and it can also be reliably obtained for challenging cases of an aggregation of aromatic and antiaromatic molecules in solution. This computational analysis is performed for the complex of coronene and an antiaromatic model compound in acetonitrile by employing the GIAO-B3LYP-PCM approach combined with a saturated basis set. Predicted ^1^H CS values are used to generate volumetric data, whose properties are thoroughly investigated. The ^1^H CS isosurface, corresponding to a value of the proton chemical shift taken from a previous experimental study, is described. The presence of the ^1^H CS isosurface should be taken into account in deriving structural information about supramolecular hosts and their encapsulation of small molecules.

## 1. Introduction

Recently, significant progress was achieved in various areas of supramolecular chemistry, as exemplified by the review articles published in 2020 [1,2,3,4]. This progress has already led to numerous applications (see an extensive survey in reference [5]) and to the preparation of a number of fascinating architectures like, for example, an enantiopure “Russian doll” [6], a triple-stranded mesocate [7], and a cage compound that can be transformed into the covalent organic framework [8]. In the process of the characterization of supramolecular structures, techniques of the liquid state NMR of protons (^1^H NMR) are crucial, which were most recently outlined in reference [9]. The proton NMR chemical shift in solution is usually the most important ^1^H NMR parameter because its value for the investigated hydrogen(s) in a guest molecule generally differs between encapsulated and bulk states, allowing a straightforward analysis of the guest binding and exchange [10]. Of course, the proton chemical shifts in a host molecule may also be important for the characterization of supramolecular complexes using the ^1^H NMR methods [11]. Some experimental ^1^H NMR studies can be usefully supplemented by the quantum chemical calculations of the theoretical counterpart of the ^1^H chemical shift in solution, that is, of the ^1^H chemical shielding affected by the solvent, ^1^H CS (see the most recent review [12] and references cited therein). We thus considered it important to check the performance of state-of-the-art methods of predictions of the geometries and NMR parameters in solution when applied to key aromatic and antiaromatic compounds [13]. As expected, this performance was found to be excellent, and it enabled us to investigate accurately determined local structural effects upon the ^1^H CS data related to the complexation in the nanospace recently described by Yamashina et al. [14].

This study followed three consecutive objectives: (1.) identifying the approach that can be applied to large molecular fragments and at the same time is capable of reliably predicting the {^1^H, ^13^C} two-dimensional (2D) heteronuclear correlation (HETCOR) spectra in solution; subsequently, (2.) applying the selected approach to the ^1^H CS of both aromatic and antiaromatic compounds in order to check its predictive power in this area; and next, (3.) using this predictive power to generate large-scale volumetric data related to the binding of coronene by an antiaromatic model compound in acetonitrile. The resulting data set was then thoroughly analyzed. Significantly, the presence of the ^1^H CS isosurface was revealed in an important interval of geometries relevant for the aforementioned binding. Such isosurfaces should be considered in the process of deriving structural information about supramolecular hosts and their encapsulation of small molecules, especially in cases where X-ray diffraction (XRD) results were unavailable. The related analysis could be usefully employed in the current research on host–guest complexes (see the survey [15], and also reference [16]).

## 2. Results

### 2.1. Selection of the Computational Approach

Some time ago [17,18] we proposed an approach that could facilitate the signal assignment of 2D spectra. Our method is based on the statistical evaluation of similarity measures between experimental chemical shifts, $\delta_{X}$ and $\delta_{Y}$, and permutations of the corresponding chemical shieldings, $\sigma_{X}$ and $\sigma_{Y}$, in an investigated system of nuclei X and Y (related statistical parameters are summarized in Appendix A). Using data from the solid-state NMR measurements and from calculations of crystalline structures, this method was applied in a number of “NMR crystallography” studies, as noted by Hodgkinson in the most recent review [19]. While most of these applications concerned the {^1^H, ^13^C} HETCOR spectra, this approach was also used to describe the ^1^H–^1^H homonuclear correlations [20]. Importantly, the methodology is the same for the solid- and liquid-state 2D spectra. For many compounds, we thus tested various combinations of the density-functional theory (DFT) functionals with atomic-orbital basis sets and with a treatment of the solvent in order to find the computational strategy for an accurate representation of solution 2D NMR data (target systems are expected to contain more than 100 atoms, and hence the second-order Møller–Plesset (MP2) or higher levels of quantum chemical theory cannot be routinely applied to this problem at present). It should be realized that both the molecular geometry and the chemical shielding need to be reliably described. Based on the aforementioned testing, it is supposed that highly accurate results can be obtained by adopting the relatively cheap technique abbreviated as GIAO-B3LYP-PCM/6-311++G(2d,2p) // B3LYP-PCM/6-311++G(2d,2p) (the gauge-independent atomic orbitals (GIAO), the combination of Becke’s three-parameter and Lee–Yang–Parr DFT functionals (B3LYP), and the polarizable continuum model (PCM) of the solvent reaction field applied in calculations of magnetic properties, preceded by the B3LYP-PCM geometry optimizations, while both computations use the standard 6-311++G(2d,2p) basis set; see Materials and Methods). Examples of its application are given below for aquatolide, a frequently studied sesquiterpene lactone, and for 3,12-di(4-aminophenyl) Ni^II^ dimesitylnorcorrole, which is one of the building blocks of the antiaromatic cage from reference [14]. Table 1 collects the key parameters for an assessment of the level of agreement between experimental and simulated 2D spectra (see Equations (A1)–(A4) in Appendix A for definitions of: the theoretical chemical shifts, $\varepsilon_{X}$ and $\varepsilon_{Y}$; the standard deviations, $s_{X}$ and $s_{Y}$; the covariance, $s_{\mathrm{XY}}$; and the norm, $n_{\mathrm{XY}}$, respectively). Further details are provided in the next paragraph and in Supplementary Materials, where the optimized coordinates of aquatolide and of the diamine compound can also be found.

**Figure 1:**
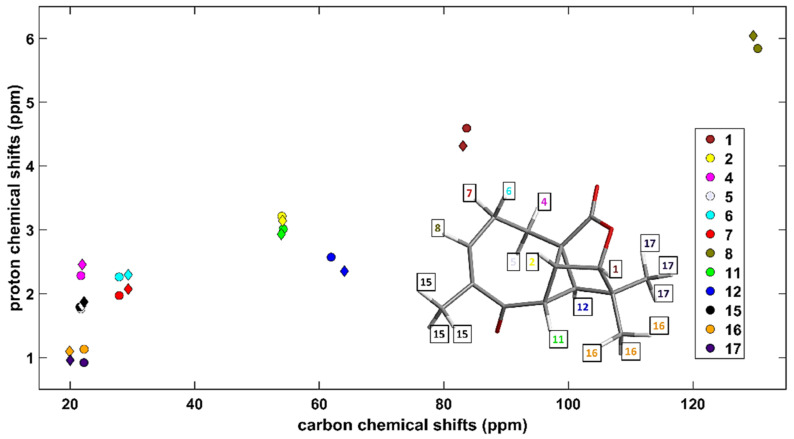
Comparison between theory and experiment for the {^1^H, ^13^C} HETCOR spectrum of aquatolide in dimethyl sulfoxide (DMSO). The measured and the GIAO-B3LYP-PCM data (see the text for details) are marked with circles and diamonds, respectively. The HETCOR pairs are specified in the inset, while their numbering is the one used in Table 1 of reference [22].

Aquatolide (C_15_H_18_O_3_) was a subject of numerous structural investigations (see the most recent study [21] and work cited therein, and also references [22,23,24]). Based on the {^1^H, ^13^C} chemical shifts taken from Supplementary Table S3 of reference [24], which were measured in DMSO (dimethyl sulfoxide), the corresponding HETCOR spectrum was simulated by the aforementioned computational approach, of course using DMSO as an implicit solvent in the PCM method. A very good agreement between experimental and predicted 2D data sets is apparent from values of the statistical parameters collected in Table 1, and from a visualization presented in Figure 1 (separate fits for all carbons and protons of aquatolide are shown in Supplementary Figures S1 and S2, while the underlying data are collected in Supplementary Tables S1–S3). Additionally, the prediction for the diamine (C_48_H_40_N_6_Ni) was highly successful, namely, of the heteronuclear single quantum correlation (HSQC) spectrum obtained in chloroform and shown in Supplementary Figure S7 of reference [14]. In particular, even small differences between experimental peak positions (<2 ppm for ^13^C and <0.2 ppm for ^1^H, see Supplementary Table S4 and also Supplementary Figure S3) were all correctly reproduced by the GIAO-B3LYP-PCM(chloroform)/6-311++G(2d,2p) calculations. It is stressed that the results would deteriorate if a different functional; a smaller basis set; an inadequate treatment of the solvent were applied.

### 2.2. The ^1^H CS in Aromatic and Antiaromatic Systems

**Figure 2:**
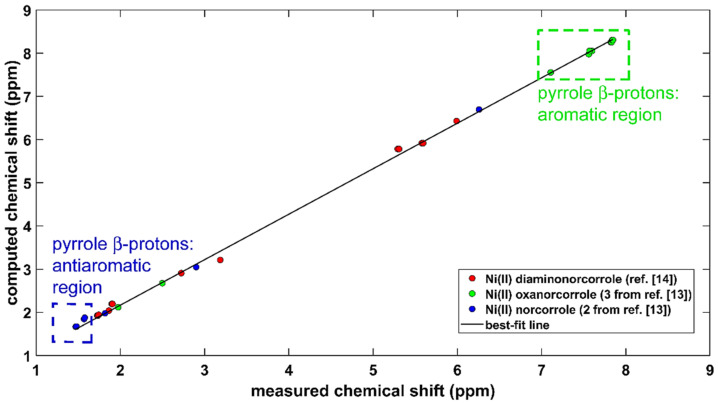
Comparison of the GIAO-B3LYP/6-311++G(2d,2p) and experimental ^1^H chemical shifts (in ppm) in chloroform of three compounds specified in the text.

The aromaticity of protons in conjugated systems was investigated for many decades, and various aspects of this topic were most recently covered in references [25,26,27]. The B3LYP-PCM-based methodology that is described in the preceding section was applied to two compounds, which are important for aromaticity/antiaromaticity studies of porphyrin analogues [13] (see the surveys [28,29]). These molecules are stable complexes of Ni^II^ with substituted norcorroles, namely, an antiaromatic Ni^II^ 6,15-dimesitylnorcorrole and aromatic Ni^II^ 6,16-dimesityl-10-oxanorcorrole. Their powerful ^1^H NMR markers of aromaticity/antiaromaticity are highlighted in Figure 2. The structurally related diamine from reference [14] (see Section 2.1) was also included in the comparison of predicted and experimental ^1^H chemical shifts, and thus the total number of investigated protons was formally equal to 100. However, because of signal averaging, the actual number of data points in the correlation is 29. They are specified in Supplementary Table S5, and the linear regression model is graphically presented in Figure 2. This model used the following simplification: the GIAO-B3LYP-PCM(chloroform)/6-311++G(2d,2p) ^1^H CS values, $\sigma_{H}$, were converted into estimates of the proton chemical shift, $\delta_{H}\prime$, using $\delta_{H}\prime =\;\sigma_{H\left(\mathrm{TMS}\right)}\;-\;\sigma_{H}$, where $\sigma_{H\left(\mathrm{TMS}\right)}$ is the ^1^H CS of a proton in tetramethylsilane (TMS). The $\sigma_{H\left(\mathrm{TMS}\right)}$ value was obtained by the same computational approach as $\sigma_{H}$, but without an inclusion of the solvent, and amounted to 31.8308 ppm. The resulting set of $\delta_{H}\prime$ values was fitted to measured chemical shifts, $\delta_{H}$, to obtain $\delta_{H}\prime$ = 1.052*$\delta_{H}$ + 0.063 ppm, and this dependence is shown as a “best-fit line” in Figure 2. A direct fit of $\sigma_{H}$ into $\delta_{H}$ has, of course, an intercept value of 31.7678 ppm (= $\sigma_{H\left(\mathrm{TMS}\right)}$ −0.063 ppm) and a slope of −1.052. Importantly, the standard deviation of the fit(s) is very small, namely, 0.070 ppm. An inspection of Figure 2 reveals that the present calculations were capable of accurately reproducing the ^1^H chemical shift changes between the proton sites in different systems throughout the whole investigated range, which also features a crowded region (from ca. 1.7 to ca. 2.0 ppm; refer to Supplementary Table S5 for the specific values).

### 2.3. Modeling the Coronene Complex

**Figure 3:**
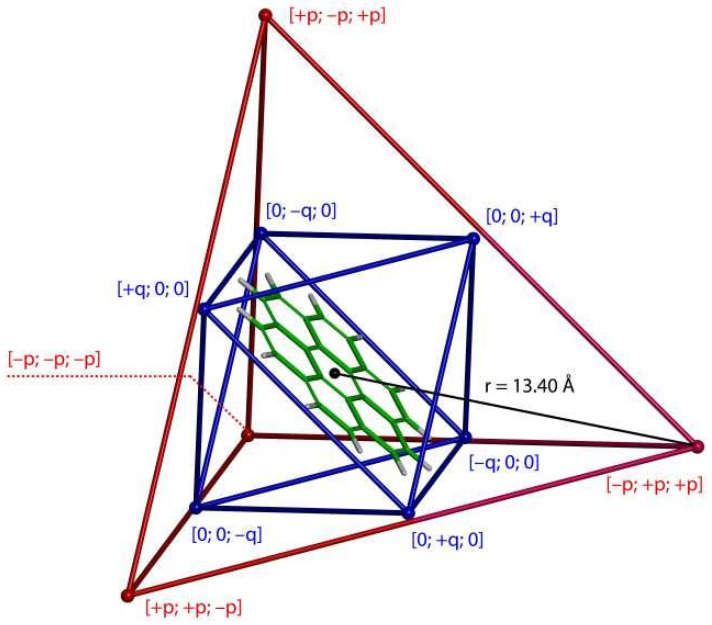
A schematic representation of the topology of a complex formed between one coronene molecule and the cage compound from reference [14]. This visualization uses the corresponding XRD geometry (CCSD refcode TOSQIE) and shows only the (Fe^+2^)_4_ (Ni^+2^)_6_ part of the cage, with coronene placed in the middle of it. The tetrahedral arrangement of Fe^+2^ centers is depicted in red ($p=\;r\;\times \;\sqrt{3}/3$, where r is the distance between the center of the cage and any of its Fe^+2^ vertices; Fe^+2^ positions are visualized as small red balls). The octahedral arrangement of Ni^+2^ centers is depicted in blue (q=c×p, where c is a constant that describes the departure of Ni from the surrounding wall; in the present case c = 0.9448; Ni^+2^ positions are visualized as small blue balls).

Nitschke and his collaborators [14] achieved a preparation of an important supramolecular system by applying the subcomponent self-assembly [30]. Their compound can be described, in short, as a cage consisting of four Fe^+2^ and six Ni^+2^ metal centers (see Figure 3), with Fe^+2^ and Ni^+2^ complexed by substituted pyridines and diaminonorcorroles, respectively. This assembly can be classified as a T3 supertetrahedron [31]: the (Fe^+2^)_4_ (Ni^+2^)_6_ framework is of *T*_d_ point-group symmetry, but the specific organic ligands and linkers lower the overall symmetry to *T*. The cage crystallizes in the cubic *I*23 space group, and its structure was solved by the single-crystal XRD analysis [14]. Nitschke et al., also used the ^1^H NMR measurements in acetonitrile to thoroughly characterize a complexation of a number of compounds inside this cage. Importantly, very high values were obtained for some complexation shifts, Δδ, Δδ = $\delta_{H\left(\prime \mathrm{bound}\prime \right)}\;-\;\delta_{H\left(\prime \mathrm{free}\prime \right)}$, where $\delta_{H\left(\prime \mathrm{bound}\prime \right)}$ and $\delta_{H\left(\prime \mathrm{free}\prime \right)}$ denote the ^1^H chemical shift of an investigated proton in the complex and in bulk, respectively. The Δδ values were positive for all guests, which was attributed to the antiaromaticity of norcorrole walls of the cage compound [14]. In the next paragraph, the computational model of the related ^1^H CS changes of a proton in coronene is described.

**Figure 4:**
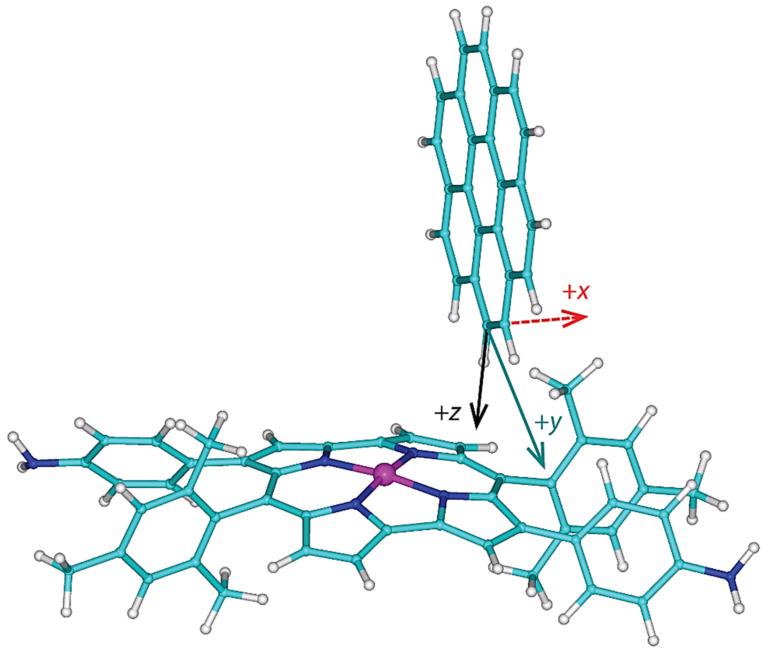
The investigated configuration of a complex formed between 3,12-di(4-aminophenyl) Ni^II^ dimesitylnorcorrole and coronene. The coordinate system depicts the dorsal (*x*), lateral (*y*), and vertical (*z*) directions discussed in the text.

It was established experimentally that the 1:2 complex had been formed between the cage and coronene molecules in acetonitrile [14]. Since the cage compound and two coronenes have 766 atoms in total, the aforementioned accurate DFT calculations cannot be currently performed for the whole assembly, and instead a reduced structural model has to be used. The present model considers one molecule of 3,12-di(4-aminophenyl) Ni^II^ dimesitylnorcorrole and one molecule of coronene, and contains 131 atoms. An initial orientation was established using the full structure of the 1:2 complex, which had been obtained by molecular mechanics (MM) calculations in reference [14] and kindly sent to one of us. The diamine and coronene, both optimized separately in acetonitrile (see Section 4 for details), were manually docked into their approximate positions in the MM structure. Obviously, the two molecules cannot be “converted” into their counterparts of the MM geometry because of discrepancies in internal coordinates due to (1) a replacement of the pyridinyl parts of the cage with protons, and (2) an application of different computational methods, B3LYP-PCM and MM, in underlying calculations. The coordinate system of the docked complex was then transformed by an appropriate translation and rotations (the related unitary quaternion was found using the INFOR program [32]). This new coordinate system is shown in Figure 4 (the coordinates are provided in Supplementary Materials “complex.xyz” file), and is convenient for creating three-parameter grids and their clear presentation. Its origin is at the carbon connected to an investigated proton. Such a proton lies at $\left[0;0;r_{\mathrm{CH}}\right]$, where $r_{\mathrm{CH}}$ is a length of the corresponding C–H bond. Thus, the *z* direction is related to the vertical distance between the diamine and coronene. The lateral offset of those two compounds changes in the *y* direction, while the *x* direction is approximately parallel to the *C*_2_ symmetry axis of the diamine; the *x* direction is called dorsal here, as it describes changes in the position of coronene “on the back” of the diamine, which are orthogonal to the lateral direction, *y* (see Figure 4). An origin of the coordinate system can in principle be positioned in any of the four possible orientations of coronene relative to two mesityl and two aniline substituents, as those substituents are dissymmetric in this respect. This ambiguity was resolved by inspecting the aforementioned MM structure. The correct quadrant of the substituted norcorrole ring was chosen, and thus the model is representative for the whole structure described in reference [14] (this model features a correct relative orientation of coronene and the diamine with respect to the removed part of the full structure).

**Figure 5:**
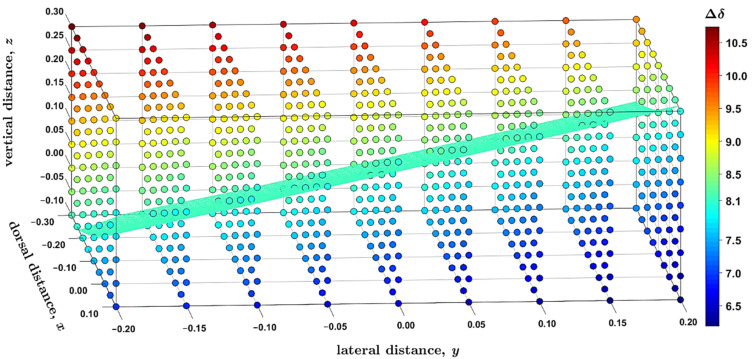
The ^1^H complexation shifts, Δδ (in ppm), at the grid of 9 × 9 × 9 distances (in Å) in the supramolecular complex described in the text. The isosurface with Δδ = 8.1 ppm is highlighted in bright cyan color whose hexadecimal string is #40FFBF.

The ^1^H complexation shifts, Δδ, were estimated by the GIAO-B3LYP-PCM calculations of the molecular fragments, namely, of an isolated coronene and of the supramolecular complex (Δδ expressed through the ^1^H CS data, $\sigma_{H}$, is Δδ = $\sigma_{H\left(\prime \mathrm{free}\prime \right)}\;-\;\sigma_{H\left(\prime \mathrm{bound}\prime \right)}$ in the notation used in Section 2.3, to reflect the sign change between the chemical shift and shielding). Importantly, the complex prepared from the MM structure has the predicted complexation shift of 7.99 ppm, almost equal to the experimental value of 8.1 ppm [14]. Hence, the Δδ landscape was surveyed in this strongly deshielded zone around the nickel-containing diamine by independently varying the three parameters pictured in Figure 4. Based on numerical experiments for multiple sets of [x; y; z] values, the final grid was cubic in shape and centered at [−0.1; 0.0; +0.1] Å, that is, at the point where the Δδ value close to 8.1 ppm had been expected (this value is in fact 7.96 ppm, as follows from Supplementary Materials “grid.txt” file). In intervals from −0.20 to +0.20 Å around this position, a uniform spacing of 0.05 Å was chosen between nine points in each dimension, leading to a dense grid of 729 points in total. Since the B3LYP-PCM(acetonitrile) calculations of the coronene–diamine complex apply as many as 2691 basis functions of the 6-311++G(2d,2p) basis set, a computation of the whole grid was rather costly (required almost 5000 computer core-days at https://metavo.metacentrum.cz/ where 16 cores of up-to-date processors were used for every grid point). However, these calculations provide fully reliable results for following changes in the ^1^H CS data with varying values of internal coordinates. Figure 5 presents those results, and its inspection reveals that the changes are large (spanning several ppm) and monotonous for all three parameters considered here. At this point it should be mentioned that non-monotonous changes were most recently described by the 2D model of the ^1^H chemical shift in certain benzene dimers [33]. In the present case, the ^1^H CS values decrease with shortening the vertical separation (which is expressed by the parameter *z* specified in Section 2.3 and visualized in Figure 4) between coronene and the diamine compound. This, of course, means that the complexation shifts increase in this direction (see Figure 5). For example, if *x* and *y* are kept fixed at −0.3 and −0.2 Å, respectively, the Δδ rises from 8.21 ppm at *z* = −0.1 Å (the H–Ni distance, *r*(H–Ni), is 3.352 Å in this geometry) to 10.74 ppm at *z* = +0.3 Å with *r*(Ni–H) = 3.037 Å. Changes in the *y* parameter can be interpreted in terms of a distance from the investigated proton to the nearest amino nitrogen, *r*(H–NH_2_). In this case, the trend is an increase in the ^1^H CS with increasing *y* values (and with concomitant shortening of *r*(H–NH_2_) distances). If, for instance, *x* and *z* are −0.3 and −0.1 Å, respectively, the Δδ drops from 8.21 ppm at *y* = −0.2 Å with *r*(H–NH_2_) = 8.545 Å to 7.35 ppm for *y* = +0.2 Å with *r*(H–NH_2_) = 8.328 Å. A variation in the dorsal direction (*x*) can be connected to changes in the distance between the investigated proton and the nearest methyl carbon of a nearby mesityl group, *r*(H–CH_3_). Namely, the ^1^H CS increases with the shortening of this distance. For instance, for *y* and *z* of −0.2 and −0.1 Å, respectively, the Δδ is 8.21 ppm at *x* = −0.3 Å with *r*(H–CH_3_) = 3.586 Å and reduces to 6.19 ppm at *x* = +0.1 Å with *r*(H–CH_3_) = 3.387 Å.

An interpolation of the results, which were provided by explicit DFT calculations at the grid points, enables one to obtain the volumetric data for even more detailed analysis of the ^1^H CS property. Hence, the aforementioned 729 grid points were interpolated by a spline using the “interp3” function of Matlab^®^. The accuracy of this interpolation was checked at three points that were randomly generated (of course, within the investigated cube). At those additional coordinates, the same calculations of the ^1^H CS were performed as for the original grid points, and results were compared to their interpolated counterparts. This comparison indicates that the interpolation was accurate to better than 0.05 ppm and created no artifacts (see Supplementary Table S6). Based on a reviewer’s comment, interpolated results obtained for the 5 × 5 × 5 points subset of the full grid are also included in Supplementary Table S6 and show that, in the present case, the outcome is essentially the same when the spacing is 0.1 Å instead of 0.05 Å. It is worth noting that measured Δδ values were rounded to 0.1 ppm anyway in reference [14]. Consequently, the interpolated data are considered to be reliable for the generation and subsequent analysis of isosurfaces within the volume investigated here. The presence of isosurfaces can certainly be anticipated on the basis of the aforementioned 2D study of benzene dimers [33], where isolines of the ^1^H chemical shift were described on some plane. However, the actual shape of the isosurface would remain unknown without searching the volume for a given isovalue. The result of such a search, which was performed using the “isosurface” function of Matlab^®^ for Δδ = 8.1 ppm, consists of 5131 points which were deposited [34] and are scrutinized in Section 4. This isosurface is graphically presented in Figure 5, showing a plenitude of internal geometries that are compatible with a value of the ^1^H complexation shift.

## 3. Discussion

**Figure 6:**
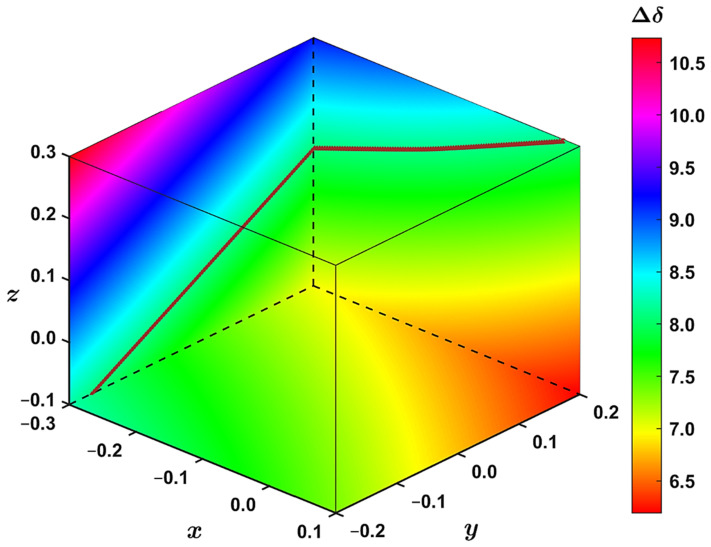
The interpolated ^1^H complexation shift, Δδ (in ppm), at slices drawn along the *x*; *y*; *z* values (in Å) discussed in the text. The brown points approximate the isosurface with Δδ = 8.1 ppm and were obtained through Equation (1).

The geometry dependence of the ^1^H CS can be easily followed using the volumetric data. In the way of an example, in Figure 6 to the left is a slice of the *yz* plane taken at *x* = −0.3 Å; at the bottom is a slice of the *xy* plane taken at *z* = −0.1 Å; to the right is a slice of the *xz* plane taken at *y* = +0.2 Å. Then, for instance, the aforementioned increase in Δδ between *z* = −0.1 Å and *z* = +0.3 Å for constant values of *x* = −0.3 Å and *y* = −0.2 Å is apparent at an area adjacent to the *z* axis of Figure 6. This figure further illustrates that the complexation shift changes monotonously with varying values of the internal coordinates. Additionally, in Figure 6, approximations to the isosurface are shown, which are described below.

It could be of interest to be able to accurately represent the isosurface for Δδ = 8.1 ppm using some simple functional form, $\Delta \delta =f\left(x,y,z\right)$, at particular ranges of the *x*; *y*; *z* parameters considered here. After a visual inspection of the vertices and some numerical experiments, it is proposed to express this isosurface by the following implicit equation (values of the coefficients $a_{1},a_{2},\ldots ,a_{10}$ are listed in Supplementary Table S7):

(1) $$\Delta \delta \left(x,y,z;\;a_{1},a_{2},\ldots ,a_{10}\right)=a_{1}x^{2}+2a_{2}xy+2a_{3}xz+2a_{4}x+a_{5}y^{2}+2a_{6}yz+2a_{7}y+a_{8}z^{2}+2a_{9}z+a_{10}=0$$

Thus, the isosurface is assumed to be a quadric surface. Its classification can be obtained by diagonalizing a symmetric 4 × 4 matrix of coefficients of Equation (1) and inspecting the signs of eigenvalues (the matrix is shown on page S7 and the resulting eigenvalues in Supplementary Table S8). In the present case, three eigenvalues are positive and one is negative. Consequently, this quadric surface is elliptic [35]. Equation (1) can be employed to immediately find an approximation to the isosurface at some constant value of *x* or *y* or *z*. Namely, after substituting such a value into Equation (1) and scanning values in one of the other two dimensions, a quadratic equation in the third parameter results. A relevant root of this quadratic equation is then an estimation of the corresponding isosurface point. Examples are shown in Figure 6 for *x* = −0.3 Å with scanning the *y* dimension and solving for *z* values, and for *y* = +0.2 Å with scanning the *x* dimension and solving for *z* values. This approximation is quite accurate (see Supplementary Figures S4 and S5).

The ^1^H CS isosurfaces may have practical implications for studies of host–guest systems, in particular, if the structural information is inferred from changes in measured ^1^H chemical shift data. The most recent examples involve details of the molecular recognition by C–H…π interactions [36], and of binding modes inside relatively small (of “yoctoliter” volume) capsules [37]. In such investigations, a possibility should be considered that fairly different position(s) of the ligand might be compatible with an experimental value of the complexation shift.

We are currently investigating how the cooperativity of binding affects the ^1^H CS values. Additional extensions of the volumetric approach may include an incorporation of the chemical shielding data of nuclei other than ^1^H. However, related ^13^C/^15^N/^17^O measurements would likely require spin-labeled samples.

## 4. Materials and Methods

Input coordinates of aquatolide, Ni^II^ 6,15-dimesitylnorcorrole, and Ni^II^ 6,16-dimesityl-10-oxanorcorrole were used as clipped out from the XRD structure with the CCSD refcode KEPZAI, YEKQUC, and YEKRAJ, respectively. Initial coordinates of 3,12-di(4-aminophenyl) Ni^II^ dimesitylnorcorrole were taken from reference [14], page S65, and symmetrized to *C*_2_. Input coordinates of coronene were prepared so that they reflect its *D*_6h_ symmetry. These structures were fully optimized in the implicit solvent specified in Section 2, and were verified to be a minimum of the respective B3LYP-PCM/6-311++G(2d,2p) potential energy surface. Subsequently, the corresponding chemical shielding calculations were carried out at the B3LYP-PCM/6-311++G(2d,2p) level with the GIAO method to overcome the gauge problem [38,39]. The Gaussian 16 program package was used [40].

## Figures and Tables

**Table 1:** Similarity measures of simulations of 2D spectra described in the text.

Parameter	Compound	
	Aquatolide	The Diamine from Reference [14]
number of ^13^C–^1^H pairs	12	8
calibration for carbons	$\varepsilon_{C}\;$= −0.92285 × $\sigma_{C}\;$+ 168.15 ppm	$\varepsilon_{C}\;$= −0.96517 × $\sigma_{C}\;$+ 176.04 ppm
standard deviation $s_{C}$	1.37 ppm	0.990 ppm
calibration for protons	$\varepsilon_{H}\;$= −0.95874 × $\sigma_{H}\;$+ 30.484 ppm	$\varepsilon_{H}\;$= −0.94376 × $\sigma_{H}\;$+ 29.951 ppm
standard deviation $s_{H}$	0.145 ppm	0.075 ppm
covariance $s_{\mathrm{CH}}$	0.013 (ppm)^2^	0.009 (ppm)^2^
norm $n_{\mathrm{CH}}$	4.55 ppm	2.62 ppm

## Data Availability

The data presented in this study are available in the article, in the Supplementary Materials, and in the Harvard Dataverse Repository [34].

## Supplementary Materials

The following are available online at https://www.mdpi.com/1422-0067/22/7/3333/s1: ‘SI.PDF’ file containing Supplementary Table S1: Data plotted in Figure 1, Supplementary Table S2: Chemical shift/shielding data for all carbons of aquatolide, Figure S1: Graphical presentation of data from Supplementary Table S2, Supplementary Table S3: Chemical shift/shielding data for all protons of aquatolide, Figure S2: Graphical presentation of data from Supplementary Table S3, Supplementary Table S4: Chemical shift/shielding data used to reconstruct the investigated HSQC spectrum, Figure S3: Graphical presentation of the investigated HSQC spectrum, Supplementary Table S5: Raw data used to prepare Figure 2, Supplementary Table S6: Comparison of the extrapolated results, Supplementary Table S7: Parameter values for Equation (1), Supplementary Table S8: Details of a diagonalization of the quadratic form from Equation (1), Figures S4 and S5: Approximated fragments of the isosurface; the file “aquatolide.xyz” with coordinates of the optimized structure of aquatolide; the file “diamine.xyz” with coordinates of the symmetrized structure of 3,12-di(4-aminophenyl) Ni^II^ dimesitylnorcorrole after the optimization in chloroform; the file “complex.xyz” with coordinates of coronene and the antiaromatic model compound; the file “grid.txt” containing the ^1^H CS data at 729 grid points.

## Appendix A

As in references [17,18], it is assumed that the measured values of the NMR chemical shifts, $\delta {\left(X\right)}_{i}$ and $\delta {\left(Y\right)}_{j}$, with i=1,…,n;j=1,…,m; and n≤m, of the nuclei X and Y forming the correlation pairs $[\delta {\left(X\right)}_{k}$; $\delta {\left(Y\right)}_{k}]$ for k=1,…,m, can be fitted to the corresponding NMR chemical shieldings, $\sigma {\left(X\right)}_{i}$ and $\sigma {\left(Y\right)}_{j}$, by minimizing

$$\underset{\delta \left(X\right);\;a,b}{\min}\;\overset{m}{\underset{k=1}{\sum }}{\left(a*\delta {\left(X\right)}_{k}+b-\sigma {\left(X\right)}_{k}\right)}^{2}$$

and

$$\underset{\delta \left(Y\right);\;c,\;d}{\min}\;\overset{m}{\underset{k=1}{\sum }}{\left(c*\delta {\left(Y\right)}_{k}+d-\sigma {\left(Y\right)}_{k}\right)}^{2}$$

in order to subsequently extract the sets, $\varepsilon {\left(X\right)}_{k}$ and $\varepsilon {\left(Y\right)}_{k}$, of so called theoretical chemical shifts, ε, from

(A1) $$\varepsilon {\left(X\right)}_{k}=a*\sigma {\left(X\right)}_{k}+b;\;\varepsilon {\left(Y\right)}_{k}=c*\sigma {\left(Y\right)}_{k}+d.$$

Within the pair-list of the above-mentioned correlation pairs, the differences between the measured and theoretical chemical shifts are given by

$$\pi {\left(X\right)}_{k}=\delta {\left(X\right)}_{k}-\varepsilon {\left(X\right)}_{k};\rho {\left(Y\right)}_{k}=\delta {\left(Y\right)}_{k}-\varepsilon {\left(Y\right)}_{k}$$

and the means by

$${\bar{\pi }}_{X}\;=\frac{1}{m}\sum_{k=1}^{m}\pi {\left(X\right)}_{k};\;{\bar{\rho }}_{Y}\;=\frac{1}{m}\sum_{k=1}^{m}\rho {\left(Y\right)}_{k}.$$

Then, the similarity of the $[\delta {\left(X\right)}_{k}$; $\delta {\left(Y\right)}_{k}]$ and $[\varepsilon {\left(X\right)}_{k}$; $\varepsilon {\left(Y\right)}_{k}]$ sets of the pairs of the chemical shifts can be quantified by the standard deviations, $s_{X}$ and $s_{Y}$,

(A2) $$s_{X}=\sqrt{\frac{1}{m-1}\sum_{k=1}^{m}{\left(\pi {\left(X\right)}_{k}-{\bar{\pi }}_{X}\right)}^{2}};\;s_{Y}=\sqrt{\frac{1}{m-1}\sum_{k=1}^{m}{\left(\rho {\left(Y\right)}_{k}-{\bar{\rho }}_{Y}\right)}^{2}}\;,$$

by the covariance, $s_{\mathrm{XY}}$,

(A3) $$s_{\mathrm{XY}}=\frac{1}{m-1}\sum_{k=1}^{m}\left(\pi {\left(X\right)}_{k}-{\bar{\pi }}_{X}\right)\left(\rho {\left(Y\right)}_{k}-{\bar{\rho }}_{Y}\right),$$

and also by the norm, $n_{\mathrm{XY}}$, that was not formally introduced previously. It is obtained simply as

(A4) $$n_{\mathrm{XY}}=\sqrt{\sum_{k=1}^{m}\pi {\left(X\right)}_{k}*\pi {\left(X\right)}_{k}+\rho {\left(Y\right)}_{k}*\rho {\left(Y\right)}_{k}}$$

and is a sum of magnitudes of all $\pi {\left(X\right)}_{k}$ and $\rho {\left(Y\right)}_{k}$ elements. In Matlab^®^ notation, if vectors “p” and “r” contain the $\pi {\left(X\right)}_{k}$ and $\rho {\left(Y\right)}_{k}$ elements, respectively, the row vector “q” is “q = [p, r]”, and a value of $n_{\mathrm{XY}}$ is obtained as “norm(q)”.
